# Peonidin-3-O-Glucoside from Purple Corncob Ameliorates Nonalcoholic Fatty Liver Disease by Regulating Mitochondrial and Lysosome Functions to Reduce Oxidative Stress and Inflammation

**DOI:** 10.3390/nu15020372

**Published:** 2023-01-11

**Authors:** Ruilin Hao, Shuhua Shan, Dandan Yang, Huimin Zhang, Yi Sun, Zhuoyu Li

**Affiliations:** 1 Key Laboratory of Chemical Biology and Molecular Engineering of National Ministry of Education, Institute of Biotechnology, Shanxi University, Taiyuan 030006, China; 2 Department of Biology, Xinzhou Normal University, Xinzhou 034000, China

**Keywords:** nonalcoholic fatty liver disease, purple corncob, peonidin-3-O-glucoside, lysosomal function, mitochondrion

## Abstract

A frequent chronic liver condition across the world is nonalcoholic fatty liver disease (NAFLD). Oxidative stress caused by lipid accumulation is generally considered to be the main cause of NAFLD. Anthocyanins can effectively inhibit the production of reactive oxygen species and improve oxidative stress. In this work, six major anthocyanins were separated from purple corncob by semi-preparative liquid chromatography. The effects of the 6 kinds of anthocyanins against NAFLD were investigated using a free fatty acid (FFA)-induced cell model. The results showed that peonidin 3-O-glucoside (P3G) can significantly reduce lipid accumulation in the NAFLD cell model. The treatment with P3G also inhibited oxidative stress via inhibiting the excessive production of reactive oxygen species and superoxide anion, increasing glutathione levels, and enhancing the activities of SOD, GPX, and CAT. Further studies unveiled that treatment with P3G not only alleviated inflammation but also improved the depletion of mitochondrial content and damage of the mitochondrial electron transfer chain developed concomitantly in the cell model. P3G upregulated transcription factor EB (TFEB)-mediated lysosomal function and activated the peroxisome proliferator-activated receptor alpha (PPARα)-mediated peroxisomal lipid oxidation by interacting with PPARα possibly. Overall, this study added to our understanding of the protective effects of purple corn anthocyanins against NAFLD and offered suggestions for developing functional foods containing these anthocyanins.

## 1. Introduction

Nonalcoholic fatty liver disease (NAFLD) is the most common chronic liver disease in the world, and its global prevalence has doubled in the past 20 years. In humans, NAFLD is characterized by excessive accumulation of lipids in the liver, which is a common complication of obesity and is related to insulin resistance and metabolic syndrome [1]. NAFLD represents a series of progressive stages of liver disease, from simple steatosis to nonalcoholic steatohepatitis that manifests as liver inflammation, necrosis, and fibrosis [2,3].

Abnormal accumulation of saturated fats such as triglycerides in the liver has been reported to be associated with the pathogenesis of NAFLD [3]. This abnormal accumulation can lead to mitochondrial dysfunction and overproduction of ROS [4]. Excessive mitochondrial ROS exacerbates mitochondrial dysfunction and can also activate NF-κB and promote tumor necrosis factor α (TNF-α), interleukin (IL)-1β, and IL-6 production and induce liver inflammation and the development of nonalcoholic fatty liver [3,4,5,6,7].

Corn originates in the American continent and is considered one of the main food sources in the world. Corn contains many bioactive compounds that provide ideal health benefits [8]. Waxy corn has a variety of grain colors, including white, yellow, red, purple, and black. The different colors of corn kernels are due to the different types and quantities of anthocyanins in the pericarp and aleurone layers. Purple corn contains more anthocyanins than other corn [9,10,11]. Purple corn anthocyanins have the potential to reduce the risk of cardiovascular disease, hypertension, obesity, diabetes, and cancer [12,13,14,15,16]. As scavengers of reactive oxygen species, anthocyanins can reduce oxidative stress [17,18]. Anthocyanins can also inhibit NF-κB and PPAR activation and IL-8 mRNA expression, improving inflammatory responses [19].

In previous studies, the anthocyanin profiles of different genotypes of purple corn in China were evaluated. The main anthocyanins of purple corn were cyanidin 3-glucoside, peonidin 3-glucoside, pelargonidin 3-glucoside, and their acylated derivatives [20,21,22]. The purpose of this study was to isolate the main components of purple corn anthocyanins, establish an NAFLD cell model induced by free fatty acids (FFAs), compare and analyze the intervention effects of different anthocyanins on NAFLD, and clarify the potential mechanisms.

## 2. Materials and Methods

### 2.1. Chemicals

HPLC-grade formic acid and acetonitrile, Nile Red, Oil Red O,3-(4,5-dimethyl-2-thiazolyl)-2,5-diphenyl-2-h-tetrazolium bromide (MTT), and NDA (naphthalene-2,3-dicar-acetaldehyde) fluorescent dyes were purchased from Shanghai Aladdin Biochemical Technology Co. (Shanghai, China). A reactive oxygen species assay kit (S0033S), DHE (Dihydroethidium), mitotracker green (C1048), mitochondrial membrane potential detection kit (C2006), Lyso-tracker Green, superoxide dismutase (SOD), glutathione peroxidation physiological enzyme (GPx), catalase (CAT) detection kit, cell lysis solution (P0013), human interleukin-1β (IL-1β) enzyme-linked immunosorbent assay kit (PI305), human interleukin-6 (IL-6) enzyme-linked immunosorbent assay kit (PI330) and human tumor necrosis factor-α (TNF-α) enzyme-linked immunosorbent assay kit (PT518) were purchased from Beyotime Biotechnology (Shanghai, China). The antibodies used in Western blot detection were purchased from Beyotime Biotechnology (Shanghai, China). An aspartate aminotransferase assay kit (C010-2-1) and alanine aminotransferase assay kit (C009-2-1) were purchased from Nanjing Jiancheng Bioengineering Institute (Nanjing, China). Other reagents used are of analytical grade.

Purple corn (Jinnuo No. 8) was obtained between August and December 2019 and was provided by the Maize Research Institute of Shanxi Academy of Agricultural Sciences (Taiyuan, China). The harvested purple corncobs were first washed with deionized water and then dried in an oven at 40 °C for 48 h until the humidity level dropped to 9.62~12.60% [23,24]. After drying, the corncob samples were ground into powder in a spice grinder (FSD-100A, Taizhou City, Zhejiang Province, China) and passed through a 100-mesh sieve.

### 2.2. Extraction and Purification of Purple Corn Anthocyanins

Anthocyanin extraction was performed according to the reference method of Li et al., with slight modifications [25]. Purple corncob samples (1000 g) were soaked at 4 °C and extracted twice in methanol (4 L) containing 0.1% HCl for 12 h each time [25]. The extract was further purified on a column of AB-8 macroporous resin (C. Chen, Somavat, Singh, and Elvira, 2017). We used an LC-16P preparative-HPLC system combined with a Shimadzu Shim-pack GIS-C18 column (20 × 250 mm, 10 μm) [26] to separate anthocyanins. The mobile phase consisted of water/acetonitrile/formic acid (80/15/5, *v*/*v*/*v*). For isocratic elution, the flow rate was 10 mL/min, the column temperature was set to 35 °C, the UV detection was set to 520 nm, and 5 mL of sample solution (15.3 g/L total anthocyanins) was injected. An automated fraction collector was used for collecting the anthocyanin fraction.

### 2.3. HPLC-DAD-MS Assay

The qualitative and quantitative analysis of C3S in samples was carried out by LCMS-8050 liquid chromatography-mass spectrometry [25]. An Agilent SB-C18 column (4.6 × 150 mm, 2.7 μm; Agilent Technologies Inc., Palo Alto, CA, USA) was used to separate the anthocyanin samples. The flow rate and injection volume were 1 mL/min and 20 μL, respectively. The column temperature was 35 °C. The chromatographic column passed through phase A (5% formic acid in water) and phase B (5% formic acid in acetonitrile). The elution gradient was as follows: 5% B (0–5 min); 5–10% B (5–10 min); 10–27.5% B (10–40 min).

The acquisition mode of MS was automatic ESI-MS/MS in positive ion mode, under the following conditions: atomizing gas flow rate, 3 L/min; heating gas flow rate, 10 L/min; interface temperature, 350 °C; DL temperature, 250 °C; heating module temperature, 400 °C; dry air flow rate, 10 L/min; fragmentation voltage, 4 KV; scan mode, Q3 Scan; scan range, 100–1000 *m*/*z*.

### 2.4. Establishment of NAFLD Cell Model

The normal human liver cell line L02 was purchased from the Type Culture Collection of the Chinese Academy of Sciences (Shanghai, China) and was cultivated in a humidified incubator at a constant temperature of 37 °C with 5% CO_2_. Ten percent fetal bovine serum, 100 units/mL penicillin, and 100 units/mL streptomycin were added to RPMI 1640 medium (Gibco). We referred to the method of Yang Xu et al. of treating L02 cells with 0.5 mM FFA to establish a NAFLD model [27]. Palmitic acid (PA) was dissolved in 0.1 M NaOH at 70 °C to obtain a 100 mM PA solution. Mix 100 mM PA solution with 100 mM oleic acid (OA) in a volume of OA/PA = 2:1. The mixture was then mixed in equal amounts with 10% BSA to obtain a 50 mM FFA stock solution. When building the NAFLD cell model, dilute the FFA stock solution to 0.5 mM with cell culture medium. The NAFLD cell model was obtained after cells were cultured for 24 h in culture medium containing 0.5 mM FFA.

#### 2.4.1. The Effect of Purple Corn Anthocyanin on Lipid Accumulation in Hepatocytes

As previously mentioned [28], the Nile Red staining procedure was used. L02 cells were seeded in a 6-well plate at a density of 1 × 10^5^ cells per well and cultivated for 24 h before being exposed to 0.5 mM FFA and treated with various concentrations of purple corncob anthocyanins (10, 100, 200, and 300 μM). The cells were then rinsed twice with PBS, treated with Nile Red (0.1 M) for 30 min at 37 °C, and observed using an IX71 inverted fluorescence microscope (Olympus, Tokyo, Japan). Only cells treated with FFA served as the FFA-treated group; the nontreated group served as the control group.

Oil Red O staining was carried out using a previously published procedure with slight modifications [29]. After the L02 cells had been washed twice with PBS and fixed for 30 min at room temperature with 4% paraformaldehyde, the supernatant was discarded. The cells were then stained with Cole’s hematoxylin solution (Solarbio Life Science CO., Beijing, China) for 5 min after being dyed with 0.6% (*w*/*v*) Oil Red O for 30 min at room temperature.

L02 cells were seeded and treated in a 6-well plate as described before. After 24 h of exposure, the cells were washed twice with 2 mL of PBS. The cells were then scraped and resuspended in PBS before being centrifuged to remove the PBS. Isopropyl alcohol was used to extract the triglycerides from the precipitated cells. The extract was centrifuged at 12,000 rpm for 10 min at 4 °C to collect the supernatant. The triglyceride content of the supernatant was determined using an assay kit (BC0625) purchased from Beijing Solbio Technology Co., Ltd. in China. The absorbance at 420 nm was measured with a Synergy HTX microplate reader (Agilent, Santa Clara, CA, USA).

#### 2.4.2. Hepatocyte Viability

L02 cells were seeded into a 96-well plate (5 × 10^3^ cells per well) and cultivated for 24 h. They were then exposed to 0.5 mM FFA for 24 h, followed by 24 h of exposure to a variety of concentrations of purple corncob anthocyanins (10, 100, 200, and 300 μM). A Infinite M200 micro-plate meter (TECAN, Männedorf, Switzerland) was used to evaluate the cell viability rate after adding 0.5 mg/mL of MTT into each well. The cell viability rate using a microplate meter (Tecan Infinite M200, Männedorf, Switzerland) at 490 nm.

#### 2.4.3. Hepatocyte Damage Detection

We tested alanine aminotransferase (ALT) and aspartate aminotransferase (AST) to investigate the damaging effect of FFA on hepatocytes and the effect of purple corncob anthocyanins [30]. L02 cells were seeded in a 6-well plate (1 × 10^5^. L02 cells were treated with FFA and different concentrations of purple corncob anthocyanins. After that, the cells were collected and washed 2 times with 0.1 mol/L phosphate buffer. The cells were then centrifuged at 1000 rpm for 10 min and the cell precipitate was collected. To the cell precipitate, 0.1 mol/L phosphate buffer was added (500 μL), and the cells were broken by ultrasound under ice water bath conditions (ultrasound power was 450 W, working 5S, pause 30S, 10 cycles). The prepared homogenate was applied to a BCA protein concentration assay kit (P0010S, Beyotime Biotechnology, Shanghai, China) to determine the protein concentration. ALT and AST activities were measured according to the aspartate and alanine aminotransferase assay kit instructions. The results are reported in units/g of protein.

### 2.5. The Effect of P3G on the Antioxidant System of Hepatocytes

#### 2.5.1. Analysis of Cellular Glutathione (GSH), Superoxide Anion Radicals (O_2_^−•^), and Reactive Oxygen Species (ROS)

Cellular ROS, O_2_^−•^, and GSH levels were measured using a ROS kit (DCF was used as the fluorescence probe), dihydroethidium (DHE), and naphtha-lene-2,3-dicar-acetaldehyde (NDA) fluorescent dyes [31,32,33]. The cells were washed twice with PBS after the treatment, and the supernatant was discarded. After that, three groups of L02 cells were stained with ROS kit (20 μM DCF probe), DHE (10 μg/mL), and NDA (50 μM), and kept at 37 °C for 30 min. After that, the stained cells were analyzed using the fluorescence microscope and FACS Canto II flow cytometry (FCM) (BD, Franklin Lakes, NJ, USA). In FCM analysis, the DCF fluorescence was excited by the laser beam (488 nm) and recorded at the FITC channel. The DHE fluorescence was determined at an excitation wavelength of 535 nm and emission of 610 nm by flow cytometry (FCM). The NDA fluorescence was recorded at the FITC channel. 

#### 2.5.2. SOD, GPx and CAT Activity Assay

L02 cells were collected with a scraper and washed once with PBS. Cell samples were lysed with cell lysis buffer and centrifuged at 12,000× *g* for 10 min at 4 °C. The supernatant was used to determine the enzyme activities of SOD, GPx, and CAT according to the (S0109 total superoxide dismutase assay kit with NBT, S0058 total glutathione peroxidase assay kit, S0051 catalase assay kit; three kits were purchased from Beyotime Biotechnology, Shanghai, China) kit instructions. 

### 2.6. The Effect of P3G on Hepatocyte Mitochondria

According to the method described by Chen et al., the mitochondrial membrane potential and mitochondrial mass were detected using the Mitochondrial Membrane Potential Detection Kit and MitoTracker Green [34,35]. L02 cells were seeded in a 6-well plate and treated as previously described. Cells were washed twice with 2 mL of PBS after 24 h of exposure. The specific steps were carried out according to the kit instructions, and the concentration of MitoTracker Green in the working solution was 100 nM. The mitochondrial membrane potential was evaluated by fluorescence microscopic imaging and FCM. The cell was stained with JC-1 for 20 min at 37 °C after 24 h of P3G treatment. In FCM analysis, fluorescence was read at 488 nm excitation and 530 nm emission for green and at 540 nm excitation and 590 nm emission for red. The ratio of aggregated JC-1 (red fluorescence) and monomeric JC-1 (green fluorescence) represented the mitochondrial membrane potential. 

The ATP level in L02 cells was also analyzed. L02 cells were seeded in a 6-well plate and treated as previously described. Following PBS aspiration, the lysate was added at a rate of 200 μL per well, and cells were lysed by repeated blowing with a pipette for 2 min. The lysed cell suspension was centrifuged for 5 min at 4 °C at 12,000 g. The assay of the ATP level in supernatant was carried out as directed by the instruction (S0026, Beyotime Biotechnology, Shanghai, China).

### 2.7. The Effect of P3G on Lysosome Function

As described previously, L02 cells were treated with FFA and different concentrations of purple corncob anthocyanins. In order to detect P3G’s effect on lysosome function, the cells were stained with Lyso-Tracker Green’s 50 nM for 30 min at 37 °C in the dark. After staining, the cells were visualized by an inverted fluorescence microscope. The results were uttered as the mean fluorescence intensity [36]. 

### 2.8. Western Blot

The cells were washed two times with PBS, gathered by a cell scraper, and lysed on ice utilizing RIPA cell lysis buffer for 30 min. The cell lysate was centrifuged at 13,000 rpm for 15 min at 4 °C. The supernatant was collected, and the protein concentration was determined. Then, equal amounts of 30 μg protein from different samples were transferred to polyvinylidene fluoride (PVDF) membranes by 10% SDS-PAGE. The membrane blocking was accomplished by using 5% skimmed milk at room temperature for 1 h. The following antibodies were used: TFEB Rabbit Polyclonal Antibody (CAT No. AF8130), Lamin B1 Rabbit Monoclonal Antibody (CAT No. AF1408), LAMP1 Rabbit Polyclonal Antibody (CAT No. AF7353), Caspase-1 Rabbit Monoclonal Antibody (CAT No. AF1681), NF-κB p65 Rabbit Polyclonal antibody (CAT No. AN365) GAPDH Rabbit Monoclonal Antibody (CAT No. AF1186) (File S1) [29].

### 2.9. The Effect of P3G on Hepatocyte Inflammation

L02 cells were planted in 6-well plates at a density of 2 × 10^5^ cells per well and incubated for 24 h. Following 0.5 mM FFA treatment, L02 cells were treated for 24 h with P3G at varied concentrations (100, 200, and 300 μM). The cell-free supernatant was obtained following P3G stimulation for 24 h by centrifuging for 5 min at 500× *g*. The ELISA method was employed to detect IL-1, IL-6, and TNF- in the supernatant according to the kit instructions (Beyotime Biotechnology, Shanghai, China). Briefly, the supernatants without dilution and cytokine standards were applied to 96-well plates covered with monoclonal antibody (coupled to HRP) and incubated at 37 °C for 30 min. The plates were then cleaned and incubated with the substrate solution for 20 min at 37 °C. The stop solution was then added, and absorbance at 450 nm was measured with a Synergy HTX microplate reader (Agilent, Santa Clara, CA, USA). The results were plotted against the standard curve’s linear component. 

### 2.10. Molecular Docking

The in vitro studies of P3G against PPARα were confirmed by computational docking studies using AutoDock Vina software [37]. From the PDB database, the PPARα’s 3D-structure program database (PDB) format file (PDB ID: 2ZNN) was obtained. Before docking simulations, the protein’s energy was minimized using the UCSF Chimera program [38]. The PyMOL software suite was used to remove the hetero atoms and water molecules [39]. Dehydrogenation, calculation of Gasteiger charges, and fusion of nonpolar hydrogen atoms were all done using the Autodock tools [40]. Finally, the PDBQT format, the only one supported by AutoDock Vina, was used to save the protein and ligand. To ensure that the active site is completely covered and that ligands can move freely, a grid box with the dimensions 29 × 29 × 29 Å was established and centered at the coordinates X = 2.91, Y = 4.87, and Z = 4.66 [41]. The number of maximum binding modes was set to 50. The docking results were visualized and analyzed using PyMOL. 

### 2.11. Molecular Dynamics Study

To gather more detailed binding information, GROMACS 2020.4 was utilized for molecular dynamics (MD) simulation. Using the acpype program, the GAFF force field was used to pre-treat P3G [41]. The amber14ffSB force field was added to charge α-glucosidase [42]. ACPYPE software was used to create the ligand topology [43]. Complexes were soaked in a cubic water box that was filled with water molecules; the distance between the complexes and the border of the box was 1 nm. The complexes’ surface charges were reduced by adding 4 Na^+^. Energy minimization was conducted. The minimized system was subjected to MD that utilized an NPT ensemble after being equilibrated by NVT at 1 bar and 300 K for 100 ps. The MD simulation was then set to run for 50 ns [44]. Root mean square deviation (RMSD), solvent accessible surface area (SASA), and hydrogen bond analyses were performed on the track and structure using VMD.

### 2.12. qRT-PCR Analysis

The expression levels of PPAR, CPT-1A, ACOX1, and actin were determined using quantitative real-time PCR testing. L02 cells were collected with a scraper from the well of the 6-well plate and washed once with PBS. Cells were lysed with 0.5 mL Trizol (Beyotime Biotechnology, Shanghai, China) and reverse transcribed to cDNA using the Prime Script RT reagent Kit (TaKaRa, Shiga, Japan) according to the manufacturer’s procedure. The primers and amplification conditions were the same as described [45]. Quantitative real-time PCR was undertaken on a CFX96 real-time PCR system (Bio-Rad Laboratories, Inc., Hercules, CA, USA), and the temperature conditions were 95 °C for 5 min, followed by 95 °C for 15 s and 60 °C for 30 s with 40 amplification cycles. The primers (5′-3′) of PPARα, CPT-1A, ACOX1, and β-actin were designed as follows: PPARα (F, 5′-TGGCTCTTGACCCTATTGG-3′; R, 5′-GGGAACAGATTTCCACATTG-3′), CPT-1A (F, 5′-CCTCCGTAGCTGACTCGGTA-3′; R, 5′-GGAGTGACCGTGAACTGAAAG-3′), ACOX1 (F, 5′-GCGGACTACACTTCATAAATGC-3′; R, 5′-CCACAGGACACCATTAAGC-3′), β-actin (F, 5′-TGGATCAGCAAGCAGGAGTA-3′; R, 5′-TCGGCCACATTGTGAACTTT-3′). 

### 2.13. Statistical Analysis

Every experiment was run at least three times, and the results were expressed as the mean ± standard deviation (SD). The fluorescence intensity and Oil Red O colored area of the photo-micrograph were analyzed using Image Pro Plus 6.0 (Media Cybernetics, Inc., Rockville, MD, USA), and the findings indicated the average fluorescence intensity and the percentage of Oil Red O colored area. One-way analysis of variance (ANOVA) was used to determine the significance between two groups, and the findings were expressed as a comparison to the results obtained for Duncan’s multiple range test or Student’s *t*-test using GraphPad Prism version 8.0.0 (GraphPad Software, San Diego, CA, USA). A *p*-value less than 0.05 was regarded as significant.

## 3. Results and Discussion

### 3.1. Extraction and Purification of Purple Corn Anthocyanins

**Figure 1:**
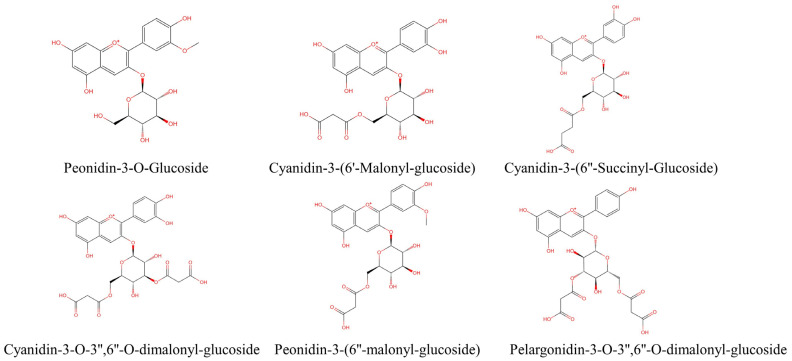
Structural formulae of anthocyanins isolated from purple corncob.

The methanol extract of purple corncob (containing 0.1% HCl) was analyzed by HPLC, and six anthocyanin peaks are shown in Figure S1. As shown in Figure S2, six anthocyanin components were collected from purple corncob extract by preparative HPLC, which were P1, P2, P3, P4, P5, and P6 peaks. Then, HPLC-DAD-ESI-MS was used for structure identification and purity determination (Figure S3). The main anthocyanins of 6 peaks were peonidin 3-o-glucoside (P3G, P1, peak 1); cyanidin 3-(6′-malonylglycoside) (C3M, P2, peak 2); cyanidin 3-(6″-succinylglucoside) (C3S, P3, peak 3); cyanidin 3-(3″,6′-dimalonylglucoside) (C3D, P4, peak 4); peonidin 3-(6′-malonylglycoside) (P3M, P5, peak 5); and pelargonidin 3-(3″,6″-dimalonylglucoside) (Pg3D, P6, peak 6) (Figure 1). Detailed identification information is provided in Table 1.

### 3.2. Purple Corn Anthocyanin Improved NAFLD Cell Model

#### 3.2.1. Purple Corn Anthocyanin Reduced the Accumulation of Lipids in NAFLD Cells

**Figure 2:**
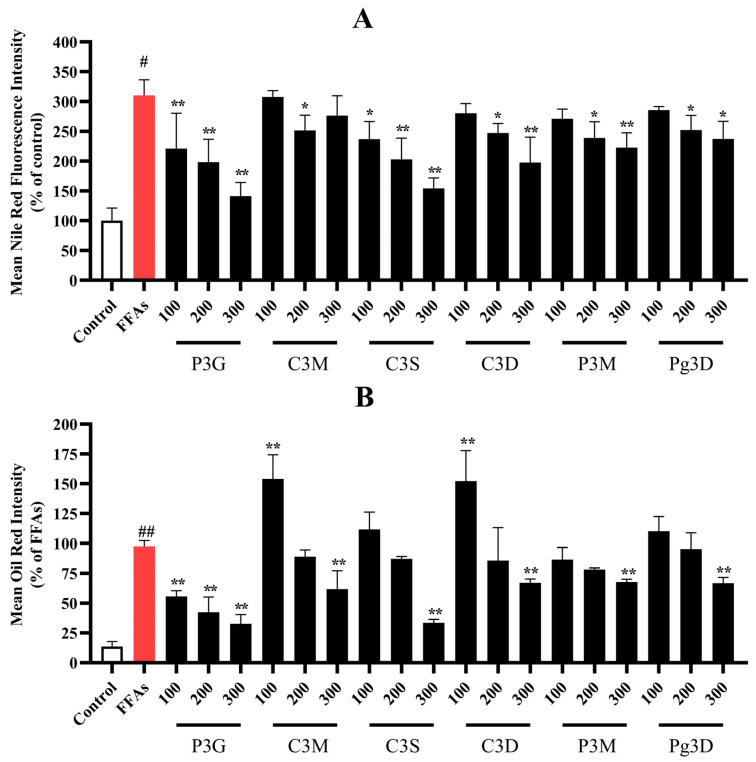
Effects of purple corncob anthocyanins on FFA-induced lipid accumulation in L02 cells via Nile Red staining and Oil Red O staining (quantitative data of panel). (**A**) Quantitative data of Nile Red staining panel, control group was considered as 100%; (**B**) quantitative data of Oil Red O staining panel, FFA-treated group was considered as 100%. (# *p* < 0.05, ## *p* < 0.01 compared with control; * *p* < 0.05, ** *p* < 0.01 vs. FFA-treated group.) P3G, peonidin-3-o-glucoside; C3M, cyanidin-3-(6′-malonylglucoside); C3S, cyanidin 3-(6″-succinyl-glucoside); C3D, cyanidin 3-o-3″,6″-o-dimalonylglucoside; P3M, peonidin 3-(6″-malonyl-glucoside); Pg3D, pelargonidin 3-o-3″,6″-o-dimalonylglucoside.

As shown in the results in Figure 2A and Figure S4, the quantitative fluorescence intensity of the control group was 100%, and the fluorescence intensity of cells in the model group induced by 0.5 mM FFA increased to 310.54 ± 26.14%. After 300 μM purple corn anthocyanin intervention, the fluorescence intensity decreased to 141.54 ± 23.07% (P3G), 276.58 ± 33.38% (C3M), 154.47 ± 17.41% (C3S), 197.88 ± 42.49% (C3D), 222.94 ± 24.83% (P3M), 237.60 ± 29.14% (Pg3D). As shown in Figure 2B and Figure S5, the same as Nile Red staining, after intervention with 300 μM purple corn anthocyanins, the area ratio of Oil Red O decreased to 32.70 ± 7.75% (P3G), 61.81 ± 15.38% (C3M), 33.56% ± 2.82% (C3S), 67.15 ± 3.09% (C3D), 67.77 ± 2.36% (P3M), 66.76 ± 4.90% (Pg3D).

**Figure 3:**
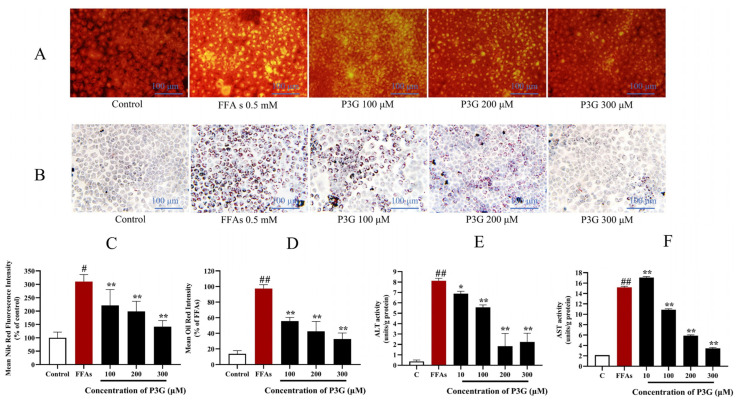
Through the use of Nile Red and Oil Red O staining, the effects of purple corn anthocyanins (P3G) on FFA-induced lipid formation in L02 cells are demonstrated. (**A**) Nile Red staining image (×200); (**B**) Oil Red O staining image (×200); (**C**) quantitative data of Nile Red-positive area in panel (**A**), control group was considered as 100%; (**D**) quantitative data of Oil Red O-positive area in panel (**B**), FFA treatment group was considered as 100%; (**E**) P3G (10, 100, 200, and 300 μM) effects on ALT activity in the presence of FFA; (**F**) P3G (10, 100, 200, and 300 μM) effects on AST activity in the presence of FFA. (# *p* < 0.05, ## *p* < 0.01 compared with control; * *p* < 0.05, ** *p* < 0.01 vs. FFA-treated group.).

The results show that purple corncob anthocyanins can effectively reduce lipid accumulation in L02 cells. Among them, P3G shows a better relief effect than other anthocyanins. As seen in Figure 3A,C, the fluorescence intensity of Nile Red dropped dramatically with increasing P3G concentrations. The fluorescence intensity was 221.30 ± 59.16 (100 μM), 198.58 ± 38.25 (200 μM), and 141.54 ± 23.07% (300 μM). The Oil Red O staining results are consistent with the Nile Red staining results (Figure 3B,D). The intracellular triglyceride content increased after FFA treatment from 9.31 ± 1.43 mmol/L (control group) to 45.83 ± 3.77 mmol/L. (Figure S6). After different concentrations of P3G treatment, the intracellular triglyceride content decreased to 44.48 ± 1.51 (10 μM P3G), 38.34 ± 1.29 (100 μM P3G), 25.18 ± 1.34 (200 μM P3G), and 15.57 ± 0.71 mmol/L (300 μM P3G). These findings suggest that P3G therapy can prevent hepatocytes from accumulating lipids as a result of FFA exposure. 

#### 3.2.2. Purple Corn Anthocyanin Enhanced the Viability of NAFLD Cell Model

The cell viability decreased significantly after the intervention of 0.5 mM FFA, which decreased to 56.33 ± 4.35% in comparison with the control group (Figure S7). The viability of the NAFLD cell model increased from 54.37 ± 3.84% (FFA) to 109.99 ± 9.48% (P3G), 88.85 ± 0.26% (C3M), 114.32 ± 11.38% (C3S), 107.82 ± 13.91% (C3D), 106.65 ± 9.33% (P3M), and 109.30 ± 5.01% (Pg3D) after treatment with six anthocyanin monomers at 300 μM. Purple corncob anthocyanin monomer has no obvious toxicity to liver L02 cells and can significantly improve the decline in cell viability caused by FFA. 

#### 3.2.3. P3G Ameliorates Hepatocyte Injury Induced by FFA

We found that FFA caused a significant increase in AST and ALT in L02 cells from 2.08 ± 0.05 units/g and 0.38 ± 0.14 units/g in the control group to 15.19 ± 0.20 and 8.13 ± 0.24 units/g, respectively. However, the P3G processing offsets the increase in AST and ALT levels (Figure 3E,F). After treatment with different concentrations of P3G, AST levels dropped to 17.08 ± 0.21 (10 μM), 10.86 ± 0.19 (100 μM), 5.87 ± 0.18 (200 μM), 3.45 ± 0.17 units/g (300 μM). The ALT levels dropped to 6.89 ± 0.23 (10 μM), 5.57 ± 0.22 (100 μM), 1.83 ± 1.22 (200 μM), and 2.24 ± 0.84 units/g (300 μM). The results showed that P3G could significantly improve hepatocyte injury induced by FFA.

### 3.3. P3G Reduces the Oxidative Stress Induced by FFA

#### 3.3.1. P3G Reduces ROS Levels in NAFLD Cell Model

**Figure 4:**
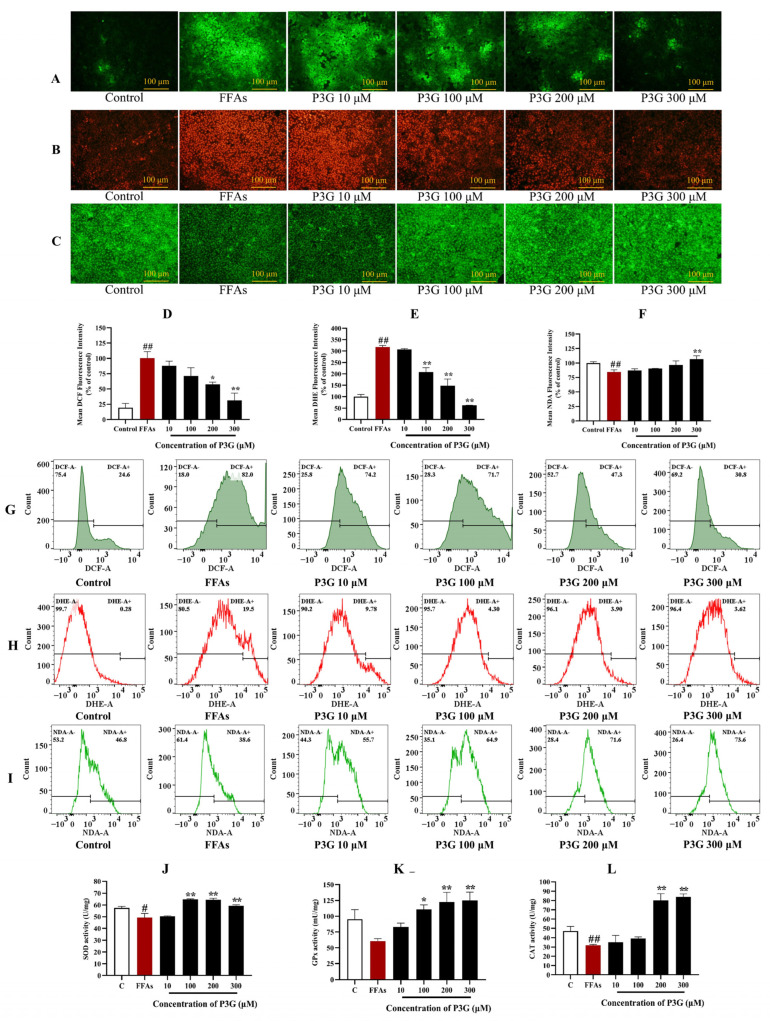
The impact of P3G on the levels of ROS, superoxide anion free radicals, GSH, and antioxidant enzyme activity in L02 cells. Following 0.5 mM FFA treatment, L02 cells were incubated with P3G at several concentrations (10, 100, 200, and 300 μM) for 24 h. Cells were then stained using the fluorescent probes DCFH-DA, DHE, and NDA after treatment. (**A**) DCF fluorescence image (×200); (**B**) DHE fluorescence image (×200); (**C**) NDA fluorescence image (×200); (**D**) quantitative data of panel (**A**), FFA-treated group was considered as 100%; (**E**) quantitative data of panel (**B**), control group was considered as 100%; (**F**) quantitative data of panel (**C**); (**G**) FCM results of DCF staining; (**H**) FCM results of DHE staining; (**I**) FCM results of NDA staining; (**J**) SOD activities; (**K**) GPx activities; (**L**) CAT activities. (The control group was considered as 100%; # *p* < 0.05, ## *p* < 0.01 compared with control; * *p* < 0.05, ** *p* < 0.01 vs. FFA-treated group).

Eliminating ROS is considered to be an effective way to alleviate NAFLD [46,47]. As shown in Figure 4A,D, 0.5 mM FFA induced a significant overproduction of ROS in L02 cells. However, P3G generated the best results at a concentration of 300 μM, and the fluorescence intensity was decreased to 31.25 ± 11.78% (compared with the FFA-treated group, *p* > 0.05) as a result of its considerable inhibitory action on the rise of ROS induced by FFA. Figure 4G shows that after FFA treatment, the percentage of DCF-positive cells increased to 82.0% compared to the control group (24.6%). After different concentrations of P3G intervention, the percentage of DCF-positive cells decreased significantly. The proportion of new cells that were DCF-positive decreased to 30.8% after 300 μM P3G treatment (Figure 4G). The flow cytometry results were consistent with the fluorescence imaging statistical results. 

Similar results were obtained from the observation of O_2_^−•^. As shown in Figure 4B,E, compared to the control group, the O_2_^−•^ level significantly increased after FFA (317.75 ± 6.47%). Flow cytometry analysis revealed that the percentage of DHE-positive cells increased to 19.5% after FFA treatment compared to 0.28% in the control group (Figure 4H). After treatment with different concentrations of P3G, the percentage of DHE-positive cells was significantly reduced in a dose-dependent manner. Treatment with 300 μM P3G reduced the percentage of DCF-positive neoplastic cells to 3.62%. O_2_^−•^ production was dose-dependently decreased by P3G administration. 

GSH is a significant endogenous nonenzymatic antioxidant that can scavenge ROS. The changes in GSH content in L02 cells were detected by NDA staining. As shown in Figure 4C,F, the GSH concentration in the FFA treatment group dropped to 84.67 ± 3.60% compared to the control group. P3G significantly increased the GSH concentration in cells (Figure 4C,F). FFA treatment reduced the proportion of NDA-positive cells from 46.8% (the control group) to 38.6%, as shown by the flow cytometry analysis results (Figure 4I). NDA-positive cells increased to 55.7% (10 μM P3G), 64.9% (100 μM P3G), 71.6% (200 μM P3G), and 73.6% (300 μM P3G) after intervention with different concentrations of P3G. The level of oxidative stress induced by FFA in hepatocytes can be efficiently decreased by P3G. 

#### 3.3.2. P3G Enhances the Activities of SOD, GPx and CAT

As shown in Figure 4J–L, FFA significantly decreased the functions of GPx and CAT in L02 cells (*p* > 0.05). SOD, GPx, and CAT activity in the 300 μM P3G treatment group increased from 49.22 ± 3.56, 60.55 ± 4.08, and 31.92 ± 0.94 u/mg to 59.24 ± 0.88, 125.19 ± 13.11, and 84.01 ± 3.13 u/mg, respectively, when compared to FFA treatment. P3G can reduce the level of oxidative stress caused by FFA by increasing the activities of SOD, GPx, and CAT.

### 3.4. P3G Strengthened the Mitochondrial Function

**Figure 5:**
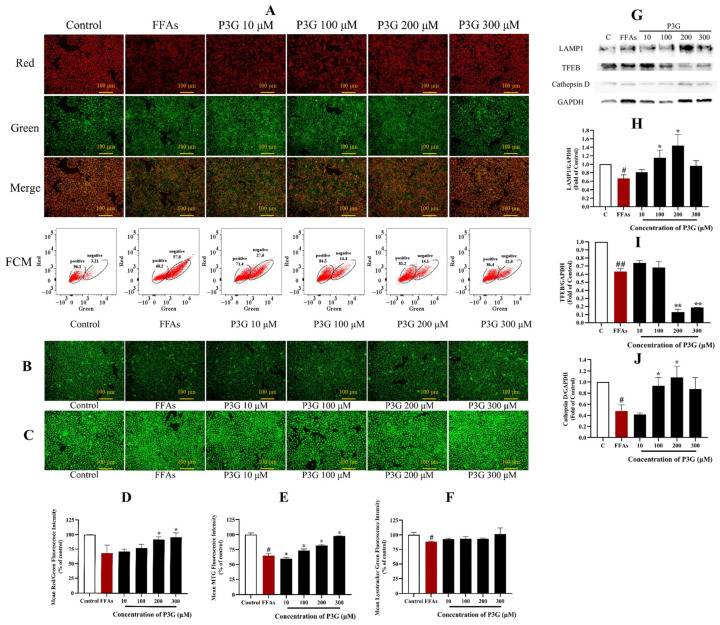
P3G’s effect on mitochondrial membrane potential, mitochondrial mass, lysosome level, and the expression of lysosome-related proteins in L02 cells. (**A**) JC-1 fluorescent image (×200) and FCM results of JC-1 staining (positive, cells with red fluorescence; negative, cells with green fluorescence); (**B**) MitoTracker Green fluorescent image (×200); (**C**) LysoTracker Green fluorescence image (×200); (**D**) quantitative data of panel (**A**); (**E**) quantitative data of panel (**B**); (**F**) quantitative data of panel (**C**); (the control group was considered as 100%; # *p* < 0.05, ## *p* < 0.01 compared with control; * *p* < 0.05, ** *p* < 0.01 vs. FFA-treated group). (**G**) Western blots of cellular cathepsin D, TFEB, and LAMP1 proteins; (**H**) quantitative data of LAMP1; (**I**) quantitative data of TFEB; (**J**) quantitative data of cathepsin D. The Western blots test was repeated 3 times and the image presented was typical of these 3 independent tests. (The control group was considered as 1.0; # *p* < 0.05, ## *p* < 0.01 compared with control; * *p* < 0.05, ** *p* < 0.01 vs. FFA-treated group).

Cell aging and apoptosis may result from the aberrant energy metabolism and mitochondrial structure caused by oxidative stress [48]. In this work, the mitochondrial membrane potential and mitochondrial mass of L02 cells were measured using JC-1 and MitoTracker Green, respectively (Figure 5). 

Figure 5A,D demonstrates that 0.5 mM FFA decreases the JC-1 fluorescence fusion result from 100% in the control group to 68.36 ± 6.63% in the experimental group. In a dose-dependent manner, P3G restored the decline in mitochondrial membrane potential. The mitochondrial membrane potential rose with an increase in P3G concentration, reaching values of 71.06 ± 4.27% (10 μM), 77.08 ± 6.71% (100 μM), 91.91 ± 4.85% (200 μM), and 96.12 ± 7.66% (300 μM). The results of the flow cytometry analysis showed that the FFA group had a significantly lower red/green ratio than the control group, while P3G reversed this trend (Figure 5A). After treatment with different concentrations of P3G, the percentage of negative cells (cells with green fluorescence) was reduced to 27.0% (10 μM P3G), 14.4% (100 μM P3G), 14.1% (200 μM P3G), and 12.8% (300 μM P3G). The mitochondrial membrane potential induced by FFA in hepatocytes was effectively increased by P3G. The mass study of mitochondria revealed the same conclusion. The fluorescence intensity of Mito-Tracker Green increased following treatment with various doses of P3G, rising from 64.673.33% (FFA) to 72.862.91% (100 μM P3G), 81.771.27% (200 μM P3G), and 97.420.43% (300 μM P3G) (Figure 5B,E). The findings demonstrate that P3G can promote mitochondrial dysfunction caused by FFA. According to Figure S8, the ATP level in the FFA-treated group was significantly lower than that in the control group (*p* < 0.01). When compared to the FFA-treated group, intervention with different doses of P3G significantly reversed ATP levels in FFA-exposed L02 cells (*p* < 0.05).

### 3.5. P3G Enhances the Lysosomal Function

In rodents with NAFLD, the lysosomal function was inhibited [49]. The fluorescence intensity of LysoTracker in the FFA-treated group reduced to 87.99 ± 1.03% compared to the control, as illustrated in Figure 5C,F. The fluorescence intensity of Lyso Tracker Green increased upon treatment with various doses of P3G, reaching 92.53 ± 1.58 (10 μM), 93.31 ± 3.73 (100 μM), 92.97 ± 1.63 (200 μM), and 101.21 ± 10.60% (300 μM).

Lysosome-associated membrane protein-1 (LAMP1) is one of the main protein components on the lysosomal membrane [50,51]. The LAMP1 level in the FFA-treated L02 cells reduced (0.67 ± 0.09) in comparison to the control group, according to the Western blot results in Figure 5G,H, but P3G considerably increased the LAMP1 level. Following treatment at different concentrations of P3G, the levels of LAMP1 increased to 0.81 ± 0.06 (10 μM), 1.15 ± 0.18 (100 μM), and 1.44 ± 0.26 (200 μM). 

The majority of lysosomal hydrolases contain cathepsin D, which is a member of the cathepsin protease family [52]. Studies have found that decreased liver cathepsin D levels in patients with NFALD will affect lysosomal acidification, resulting in the accumulation of lipids. The findings of the verification of the cathepsin D expression level are displayed in Figure 5G,J. The results show that the expression level of cathepsin D increased to 1.08 ± 0.20 after treatment with 200 M P3G, almost matching the expression level in the control cells. These results suggest that P3G intervention can boost lysosomal function, up-regulate LAMP1 and cathepsin D expression, and promote lysosomal biosynthesis.

TFEB is the basic transcription factor helix-loop-helix leucine zip from the NITF family [53]. TFEB is the main transcriptional regulator of autophagy and lysosomal biogenesis. Overexpression of TFEB has been reported to produce improved autophagy, which in turn helps to break down accumulated lipids in liver cells to treat various genetic and dietary models of obesity and alcoholic liver disease [54,55,56]. In this work, we examined whether P3G-induced lysosomal function in L02 cells involves TFEB. The expression of the cytoplasmic TFEB protein decreased following FFA treatment (0.63 ± 0.04), as shown in Figure 5G,I. The expression of TFEB in the cytoplasm further decreased after treatment with 300 μM P3G (0.19 ± 0.01), whereas the expression in the nucleus dramatically increased (1.18 ± 0.05). P3G treatment did not significantly increase the expression of total TFEB in cells but significantly promoted the translocation of TFEB to the nucleus. 

### 3.6. P3G Reduces the Level of Inflammation in NAFLD

**Figure 6:**
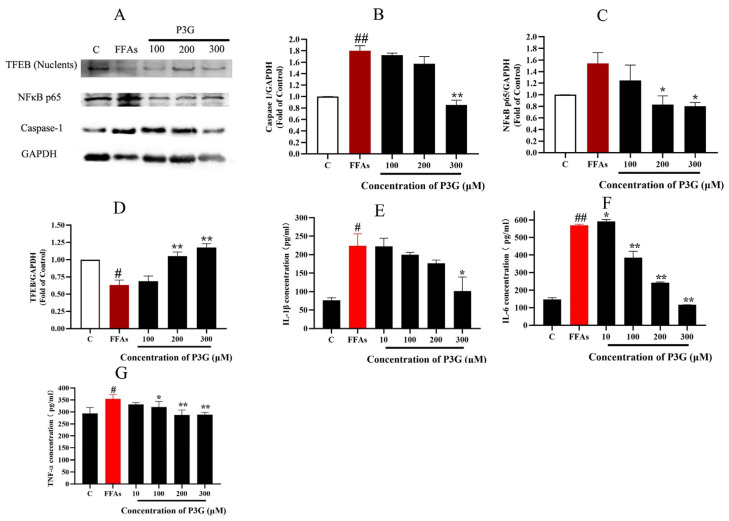
Effect of P3G on the expression of inflammation-related proteins in L02 cells. Following 0.5 mM FFA treatment, L02 cells were incubated with P3G at various doses (100, 200, and 300 μM) for 24 h. (**A**) Western blots of cellular caspase-1, NF-κB p65, and TFEB (Nuclents) proteins; (**B**) quantitative data of caspase-1; (**C**) quantitative data of NF-κB p65; (**D**) quantitative data of TFEB (Nuclents); (the Western blots test was repeated 3 times and the image presented was typical of these 3 independent tests. The control group was considered 1.0; # *p* < 0.05, ## *p* < 0.01 compared with control; * *p* < 0.05, ** *p* < 0.01 vs. FFA-treated group). The amount of IL-1β (**E**), IL-6 (**F**), and TNF-α (**G**) were determined according to the ELISA product instructions; (**E**) quantitative data of IL-1β; (**F**) quantitative data of IL-6; (**G**) quantitative data of TNF-α. (Control group was considered as 1.0; # *p* < 0.05, ## *p* < 0.01 compared with control; * *p* < 0.05, ** *p* < 0.01 vs. FFA-treated group.).

The rate of inflammation in NAFLD increases with the build-up of ROS [57,58,59]. Caspase-1 is an important target of inflammasomes, which in turn increases cytokines such as interleukin-1β and tumor necrosis factor α (TNFα) and promotes the development of inflammation in NAFLD. In rodent models of NAFLD, the development of hepatic inflammation has also been associated with increased NF-κB activity [60]. In the NAFLD model, increased stimulation of ROS promotes NF-κB expression and the development of inflammation [61,62]. The inflammatory cytokines IL-1, IL-6, and TNF-α were tested by ELISA, and the effects of FFA and P3G on the expression of caspase-1 and NF-B were examined by Western blotting. The result is shown in Figure 6. FFA treatment significantly increased the expression of caspase-1, reaching 1.80 ± 0.084 compared to the control group. After 300 μM P3G treatment, the level of caspase-1 decreased to 0.85 ± 0.082 in comparison with the control group. P3G significantly reduced the expression levels of activated NF-κB to 1.24 ± 0.27 (100 μM), 0.83 ± 0.15 (200 μM), and 0.80 ± 0.06 (300 μM) (Figure 6). At the same time, according to the results of Figure 6, P3G significantly reduced the production of IL-6, TNF-α, and IL-1β and reduced the inflammation caused by FFA.

**Figure 7:**
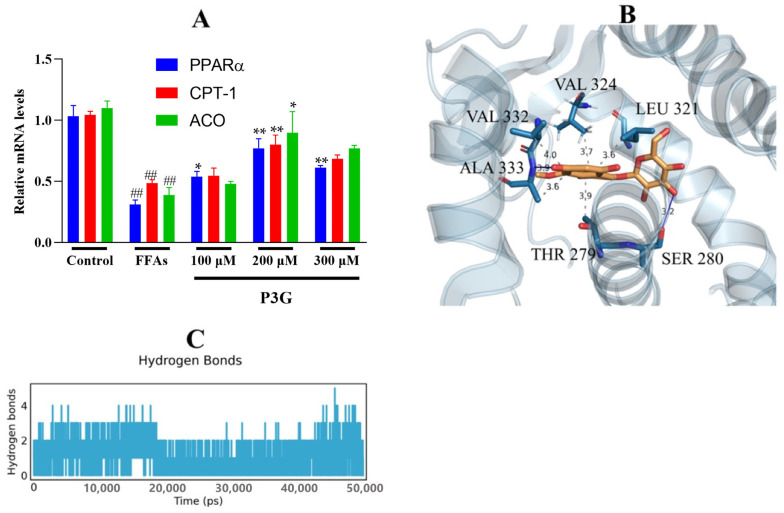
P3G’s effects on the expression level of PPAR and associated enzymes, as well as the result of their molecular docking. Following 0.5 mM FFA treatment, L02 cells were incubated with P3G at various doses (100, 200, and 300 μM) for 24 h. (**A**) The mRNA level of PPARα, CPT1A, and ACOX1 (Control group was considered as 1.0; ## *p* < 0.01 compared with control; * *p* < 0.05, ** *p* < 0.01 vs. FFA-treated group.); (**B**) the predicted binding complex of P3G and PPAR, P3G is colored in yellow, hydrogen bonds are colored in blue, and π−σ hydrophobic interactions are colored in gray; (**C**) with increasing simulation time, the amount of hydrogen bonds between P3G and PPAR dynamically changes.

As a transcription factor, TFEB promotes the expression of PPARα [63]. We found that P3G significantly promoted TFEB expression, which in turn ameliorated lysosomal inhibition during NAFLD. Based on this finding, we further explored P3G’s effects on the expression of PPARα and the peroxisomal genes CPT1A and ACOX1. Figure 7 demonstrates that the expression of PPARα, ACOX1, and CPT1A, were downregulated in L02 cells treated with FFAs. P3G upregulates the expression of PPARα protein and downstream target proteins ACOX1 and CPT1A in a dose-dependent way (Figure 7), which in turn promotes peroxisomal degradation of aberrantly accumulated fatty acids via β-oxidation. Yaoyao Jia et al. found that cyanidin-3-O-glucoside, pelargonidin-3-O-glucoside, and delphinidin-3-O-glucoside not only induce PPAR gene expression but also exert hypolipidaemic by activating PPARα activity [64]. Based on these results, we postulated that there might exist potential intermolecular interactions between PPARα and P3G; a molecular docking analysis was therefore carried out. Figure 7C depicts the interactions between P3G and PPARα amino acid residues. Hydrophobic interactions and hydrogen bonds are the main forces between P3G and PPARα. P3G forms hydrogen bonds with bond lengths of 3.2 and 3.9 Å with residues SER 280, ALA 333 of PPARα, respectively. P3G binds to THR 279, LEU 321, VAL 324, VAL 332, and ALA 333 of PPARα, respectively, to form σ–π bonds with bond lengths of 3.9, 3.6, 3.7, 4.0, and 3.6 Å. The MD analysis showed that the number of hydrogen bonds finally stabilized at 2 during the simulation, which was consistent with the docking results (Figure 7D). 

### 3.7. Molecular Dynamics

Molecular dynamics (MD) simulations supply elaborate information between proteins and ligands at different time scales under simulated physiological conditions, which helps us further understand the binding process [65]. As shown in Figure S9, the potential energy, temperature, pressure, and density of PPARα and PPARα/P3G complexes reached a steady state in a short time during the MD simulation. This result indicates that the topology files of protein and ligand and the parameters implied in the MD simulation process are reasonable.

**Figure 8:**
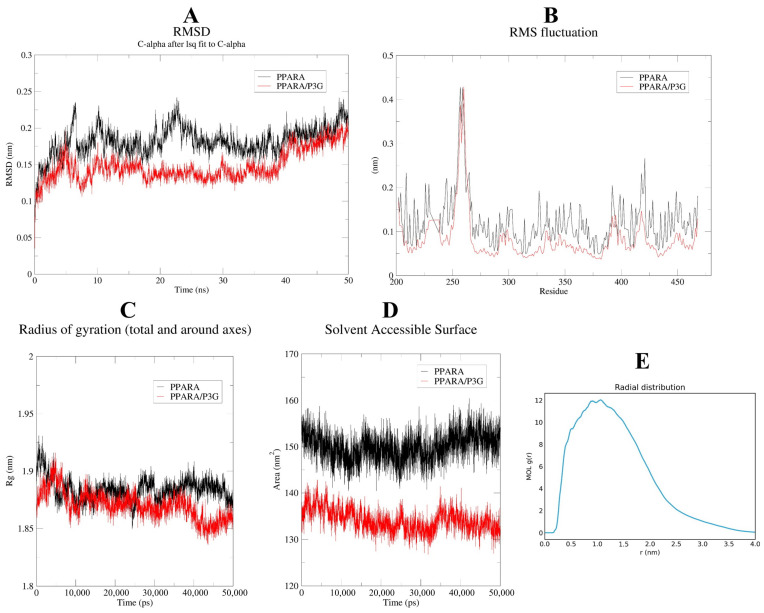
MD simulation of P3G with PPARα for 50 ns. The RMSD (**A**), RMSF (**B**) plots, and radius of gyration versus time graph (**C**), complexes solvent-accessible surface area of the PPARα-P3G complex and free PPARα backbone (**D**); (**E**) radial distribution between protein and ligand.

**Figure 9:**
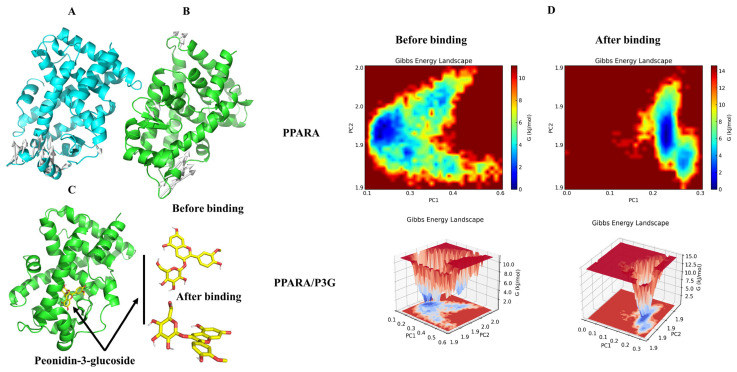
The changes of structure and Gibbs energy landscape in PPARA and PPARA/P3G complex before and after MD simulations. (**A**) Superimposition of the structures of PPARA pre-MD and post-MD simulations, white arrows indicate structures that have changed before and after MD; (**B**) superimposition of the structures of PPARA from PPARA/P3G complex pre-MD and post-MD simulations, white arrows indicate structures that have changed before and after MD; (**C**) 3D binding graph of P3G with PPARA and conformational changes of P3G; (**D**) the Gibbs energy landscape plot obtained during 50 ns MD simulations for free PPARA and PPARA/P3G complex.

To determine when the steady state will arrive and to verify the stability of the simulation, the root mean square deviation (RMSD) is used [66]. Figure 8A demonstrates that after 50 ns of simulation, free PPAR’s ultimate RMSD value is 0.2 nm. In contrast, the RMSD value of the PPARα/P3G complex showed a relatively stable state at about 0.18 nm at the end of the simulation time. It may be due to the formation of hydrogen and hydrophobic bonds between P3G and PPARα, which leads to a more stable conformation of the complex. The root mean square fluctuations (RMSF) values were also calculated for all residues on all time scales (Figure 8B). The remaining sites were comparatively constant compared to free PPARα, with the exception of the significant fluctuations in residues 300–400 in the complex system, indicating that P3G triggered flexible alterations in PPARα residues 300–400. Interestingly, we found that the radius of gyration of the complex was smaller than that of the free PPARα (Figure 8C), suggesting that the binding of P3G pushed the protein to become more compact. The binding of P3G also reduced the solvent-accessible surface area (SASA) of the complex (Figure 8D). The radial distribution function (RDF) is usually used to reveal the distribution density of ligands at a certain distance (r) from the central atom of the protein [67]. Figure 8E shows the RDF of P3G with a typical peak at 1 nm, representing the average distance between the central atom of the protein and P3G, after which a sharp decrease in g(r) is noted when r(nm) is large. The structural alterations in the protein and ligand before and after P3G’s binding to PPAR were also looked at, along with changes in the Gibbs energy landscape profiles. The distinction between PPARα and PPARα/P3G complexes before and after MD simulations is shown in Figure 9A,B. Figure 9A,B indicate that, when P3G binds to PPARα, the structure protein changes significantly in the P3G’s binding area. After the simulation, Figure 9C depicts a sizable modification in the spatial structure of P3G. The change of PPARα with P3G after MD simulation may be due to the stronger interaction between P3G and PPARα. The change of PPARα and P3G after MD simulation may be due to the stronger interaction between P3G and PPARα. The free energy shape of PPARα and PPARα/P3G complexes is depicted in Figure 9D. (both 2D and 3D landscapes). Figure 9D shows that the free energy distribution of PPARα/P3G is more concentrated than that of free PPARα, demonstrating that P3G promotes the conformation of PPARα to become more compact and stable.

The secondary structures of the PPARα and PPARα/P3G complexes as well as Ramachandran plots were also examined. Figure S10 and Video S1 indicates how the distribution of PPARα’s secondary structures underwent a significant change after the binding of P3G, particularly the secondary structure formed by the amino acid residues at positions 330–340. After P3G was included, the secondary structure of the amino acid residues at positions 330 to 340 altered from S BEND to 3-Helix. The Ramachandran plot of ALA333 changed from 60°, 60° (phi, psi angle) to −60°, 120° (phi, psi angle) after combining with P3G (Figure S10), and this shift promotes the formation of 3-Helix. 3-Helix may be more advantageous than S-BEND for maintaining the structure of the protein [68]. Due to the secondary structure transformation, the PPARα /P3G complex has a more compact conformation compared to the free PPARα (the movie provided in the Supplementary Material describes this process).

## 4. Discussion

The liver is essential for lipid metabolism [69]. When lipids accumulate, fats are primarily stored as triglycerides in hepatocytes, resulting in steatosis, the main histological feature of nonalcoholic fatty liver disease [70]. Anthocyanins are polar compounds found in foods as glycosides and acylated glycosides that have a variety of health-promoting properties. Many studies have previously reported that may have significant potential in the prevention and treatment of NAFLD [71]. Anthocyanins from Bilberry, Cherry, and Grape-Skin have been shown to improve lipid metabolism and reduce triglyceride levels in the NAFLD model [72,73,74]. We isolated six anthocyanin components from purple corn cob and tested their ability to interfere with NAFLD in this study. We discovered that P3G had the greatest ability to improve NAFLD. The development of NAFLD and oxidative stress are tightly connected [75]. When NAFLD occurs, lipid accumulation leads to mitochondrial DNA and protein abnormalities, resulting in abnormal electron transfer to form superoxide or hydrogen peroxide [76]. The activities of GSH, CAT, and other antioxidant enzymes in NAFLD patients are low, so ROS cannot be cleared in hepatocytes in time [77]. Therefore, we investigated the effect of P3G on free radicals in LO2 cells. The findings demonstrated that P3G is an effective free radical scavenger. This finding is consistent with the fact that P3G exposure increases the activity of antioxidant enzymes such as SOD, GPx, and CAT in cells. Anthocyanins from purple sweet potato and mulberry have been shown to reduce ROS production, restore glutathione (GSH) and antioxidant enzyme activity, and protect experimental mice from free radical-mediated endoplasmic reticulum stress [78,79]. 

Mitochondria are involved in the regulation of intracellular calcium levels, cellular homeostasis, and ROS [80]. Mitochondrial dysfunction and an impaired mitochondrial respiratory chain have been reported in NAFLD patients [81]. Oxidative stress causes mitochondrial dysfunction and an impaired mitochondrial respiratory chain, which further reduces cellular catabolism of lipids and energy supply [82]. This could hasten the progression of liver inflammation and NAFLD. Reducing mitochondrial dysfunction and restoring the mitochondrial respiratory chain may be an effective way to treat NAFLD. This study also confirmed that P3G, in a dose-dependent manner, reduces mitochondrial membrane damage, improves mitochondrial information, and increases ATP supply in cells. 

Lysosomes promote the degradation of polysaccharides, proteins, and lipids/free fatty acids (FFA) [83]. Lysosomes can alleviate the lipid accumulation of hepatocytes by selectively degrading triglycerides (TGs) and cholesterol [84]. Lysosomal inhibition occurs during NAFLD, and genes involved in peroxisome biogenesis and lipid metabolism are significantly downregulated following lysosomal inhibition [85]. Expression of PPARα is significantly downregulated during NAFLD [86]. Peroxisome genes such as CPT1A and ACOX1 are transcriptional targets of PPARα, and lysosomal repression, reduction in peroxisome, and fatty acid metabolism-related enzyme activities are associated with PPARα [85]. This study discovered that P3G intervention dramatically increased the expression of the lysosomal markers LAMP1 and cathepsin D. In the meantime, P3G might encourage TFEB’s nuclear translocation and promote the expression of PPARα. This outcome is consistent with Yang Xu et al.’s findings [27]. The peroxisome’s CPT1 and ACOX1 expression was further encouraged by increased PPARα expression. The results of molecular docking and molecular dynamics assays showed that the major interactions between P3G and PPAR are hydrogen bonds and hydrophobic contacts. The formation of hydrogen bonds and hydrophobic forces between P3G and PPAR facilitates the modification of PPARα’s secondary structure. The modification of the secondary structure may aid in the stabilization of the P3G/ PPARα complex and may increase the activity of PPARα.

The variation in the OH part, the quantity and type of OH part methylation, the degree of OH part methylation, and the binding position of these linkages all contribute to the structural differences of anthocyanins [87]. In comparison to the other five acyl-containing anthocyanins, the acyl-free P3G showed better effectiveness in improving NAFLD, according to this study. In comparison to the other five acyl-containing anthocyanins, the acyl-free P3G showed better effectiveness in reducing NAFLD, according to this study. Acylation may improve anthocyanin stability in aqueous solution [88], but it may also impair anthocyanin biological activity. This could be related to anthocyanin catabolism in cells. The investigation of the impact of acyl groups on the biological activity of anthocyanins will be the primary focus of the following phase of research.

## 5. Conclusions

In this study, an established hepatocyte model was used to evaluate the effect of purple corncob anthocyanin on NAFLD, in which P3G showed the potential to improve FFA-induced NAFLD. Further studies found that P3G seems to reduce FFA-induced oxidative stress by reducing mitochondrial damage, including the increase in ROS and superoxide anion levels. P3G may enhance the function of lysosomes by regulating the translocation of TFEB, so as to promote the degradation of lipid peroxide in hepatocytes and reduce the possibility of ROS production. P3G can also reduce caspase-1 and NF-κB expression to alleviate the level of hepatocyte inflammation caused by FFA. P3G interacts with PPARA primarily through hydrogen bonds and hydrophobic forces, according to the results of molecular docking and MD simulation analysis. The interaction with P3G increases the stability and compactness of the PPARA structure. P3G may upregulate the expression of genes involved in the oxidative catabolism of fatty acids by activating PPARA in addition to upregulating the expression of PPARA through TFEB, thereby reducing the aberrant buildup of fatty acids. Our research enriches the understanding of the biological function of purple corncob anthocyanins and provides ideas for the development of functional foods to improve NAFLD.

## Figures and Tables

**Table 1:** Identification of anthocyanins monomer in the purple corn cob.

	LC-ESI-MS	MS/MS	DAD	
Peaks	[M]^+^ (*m*/*z*)	Fragments [M + H]^+^ (*m*/*z*)	Vis-Max (nm)	Compounds
1	463	301	516/246/234/279/212	Peonidin-3-O-glucoside
2	535	287	517/246/234/280	Cyanidin-3-(6′-malonyl-glucoside)
3	549	287	516/246/235/280	Cyanidin-3-(6″-succinyl-glucoside)
4	621	287	517/246/280/234/	Cyanidin-3-O-3″,6″-O-dimalonyl-glucoside
5	549	301	516/246/234/280	Peonidin-3-(6″-malonyl-glucoside)
6	605	271	501/245/235/267/432	Pelargonidin-3-O-3″,6″-O-dimalonyl-glucoside

## Data Availability

Not applicable.

## Supplementary Materials

The following supporting information can be downloaded at: https://www.mdpi.com/article/10.3390/nu15020372/s1, Figure S1: High-performance liquid chromatography–diode array detector (HPLC-DAD) (520 nm) chromatogram of a crude anthocyanin extract from purple corn cob. serial numbers (1~6) correspond to peak numbers in Table 1; Figure S2: Semi-HPLC-UV/Vis (520 nm) chromatogram of a purified anthocyanin extract; Figure S3: High-performance liquid chromatography–diode array detector (HPLC-DAD) (520 nm) chromatogram of purple corncob anthocyanin monomer, serial numbers (1~6) correspond to peak numbers in Table 1; Figure S4: Effect of purple corncob anthocyanidins on FFAs-induced lipid accumulation in L02 cells via Nile Red staining. P3G, peonidin-3-o-glucoside; C3M, cyanidin-3-(6′-malonylglucoside); C3S, cyanidin 3-(6″-succinyl-glucoside); C3D, cyanidin 3-o-3″,6″-o-dimalonylglucoside; P3M, peonidin 3-(6″-malonyl-glucoside); Pg3D, pelargonidin 3-o-3″,6″-o-dimalonylglucoside. Figure S5: Effect of purple corncob anthocyanidins on FFAs-induced lipid accumulation in L02 cells via Oil Red O staining. P3G, peonidin-3-o-glucoside; C3M, cyanidin-3-(6′-malonylglucoside); C3S, cyanidin 3-(6″-succinyl-glucoside); C3D, cyanidin 3-o-3″,6″-o-dimalonylglucoside; P3M, peonidin 3-(6″-malonyl-glucoside); Pg3D, pelargonidin 3-o-3″,6″-o-dimalonylglucoside. Figure S6: The intracellular triglyceride contents in L02 hepatic cells. Following 0.5 mM FFA treatment, L02 cells were incubated with P3G at several concentrations (10, 100, 200, and 300 μM) for 24 h. Triglyceride contents were assayed by assay kits as described in the text. Values were mean ± SD (*n* = 3) and expressed in mmol/L. (The control group was considered as 100%; # *p* < 0.05, ## *p* < 0.01 com-pared with control; * *p* < 0.05, ** *p* < 0.01 vs. FFA-treated group.); Figure S7: The effect of purple corncob anthocyanin monomers on the viability of L02 cells after treated by FFAs, control group was considered as 100%. (# *p* < 0.05 compared with control, * *p* < 0.05 vs. FFAs-treated alone). P3G, peonidin-3-o-glucoside; C3M, cyanidin-3-(6′-malonylglucoside); C3S, cyanidin 3-(6″-succinyl-glucoside); C3D, cyanidin 3-o-3″,6″-o-dimalonylglucoside; P3M, peonidin 3-(6″-malonyl-glucoside); Pg3D, pelargonidin 3-o-3″,6″-o-dimalonylglucoside; Figure S8: The ATP contents in L02 hepatic cells. Following 0.5 mM FFA treatment, L02 cells were incubated with P3G at several concentrations (10, 100, 200, and 300 μM) for 24 h. ATP contents were assayed by assay kits as described in the text. Values were mean ± SD (*n* = 3) and expressed in nmol/L. (The control group was considered as 100%; # *p* < 0.05, ## *p* < 0.01 com-pared with control; * *p* < 0.05, ** *p* < 0.01 vs. FFA-treated group.); Figure S9: Evaluation of MD simulations. (A) The potential energy changes during energy minimization. (B) The temperature changes during NVT simulation. Pressure (C) and density (D) changes during NPT simulation; Figure S10: Ramachandran plot and secondary structure of PPARA and PPARA/P3G complex. File S1: Raw western blot bands. Video S1: Dynamic trajectories of proteins and ligands during the simulation.
