# Supplementary material for: Peonidin-3-O-Glucoside from Purple Corncob Ameliorates Nonalcoholic Fatty Liver Disease by Regulating Mitochondrial and Lysosome Functions to Reduce Oxidative Stress and Inflammation
Source: Nutrients. 2023 Jan 11;15(2):372. doi: 10.3390/nu15020372 (PMC9866220; doi:10.3390/nu15020372)
Supplement: Supplementary file 1 [file nutrients-15-00372-s001.zip › nutrients-2085833-supplementary figures.pdf]

Supplementary Material

Supplementary Figures

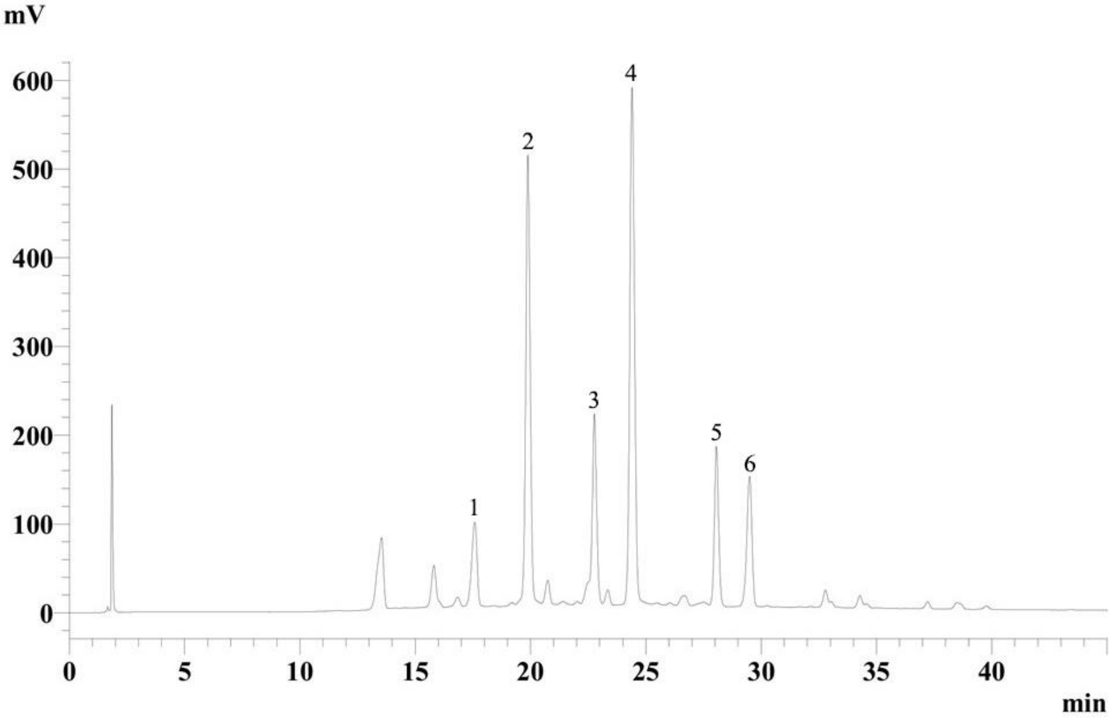

Figure S1. High-performance liquid chromatography–diode array detector (HPLC-DAD) (520 nm) chromatogram of a crude anthocyanin extract from purple corn cob. serial numbers (1~6) correspond to peak numbers in Table 1.

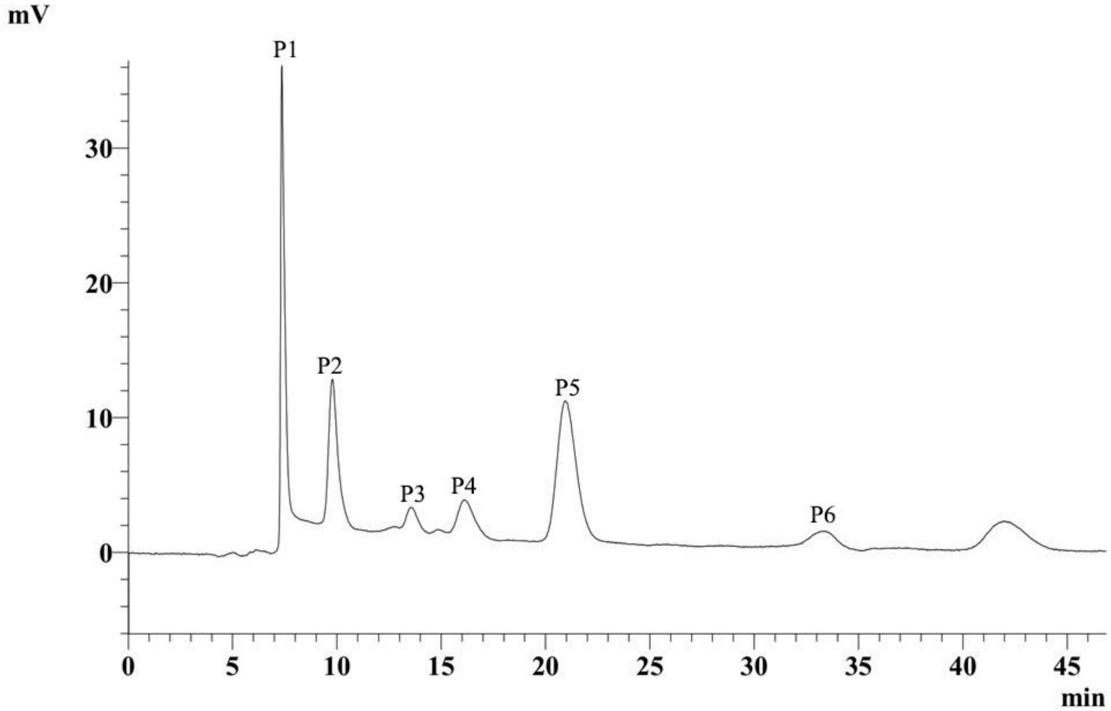

Figure. S2. Semi-HPLC-UV/Vis (520 nm) chromatogram of a purified anthocyanin extract.

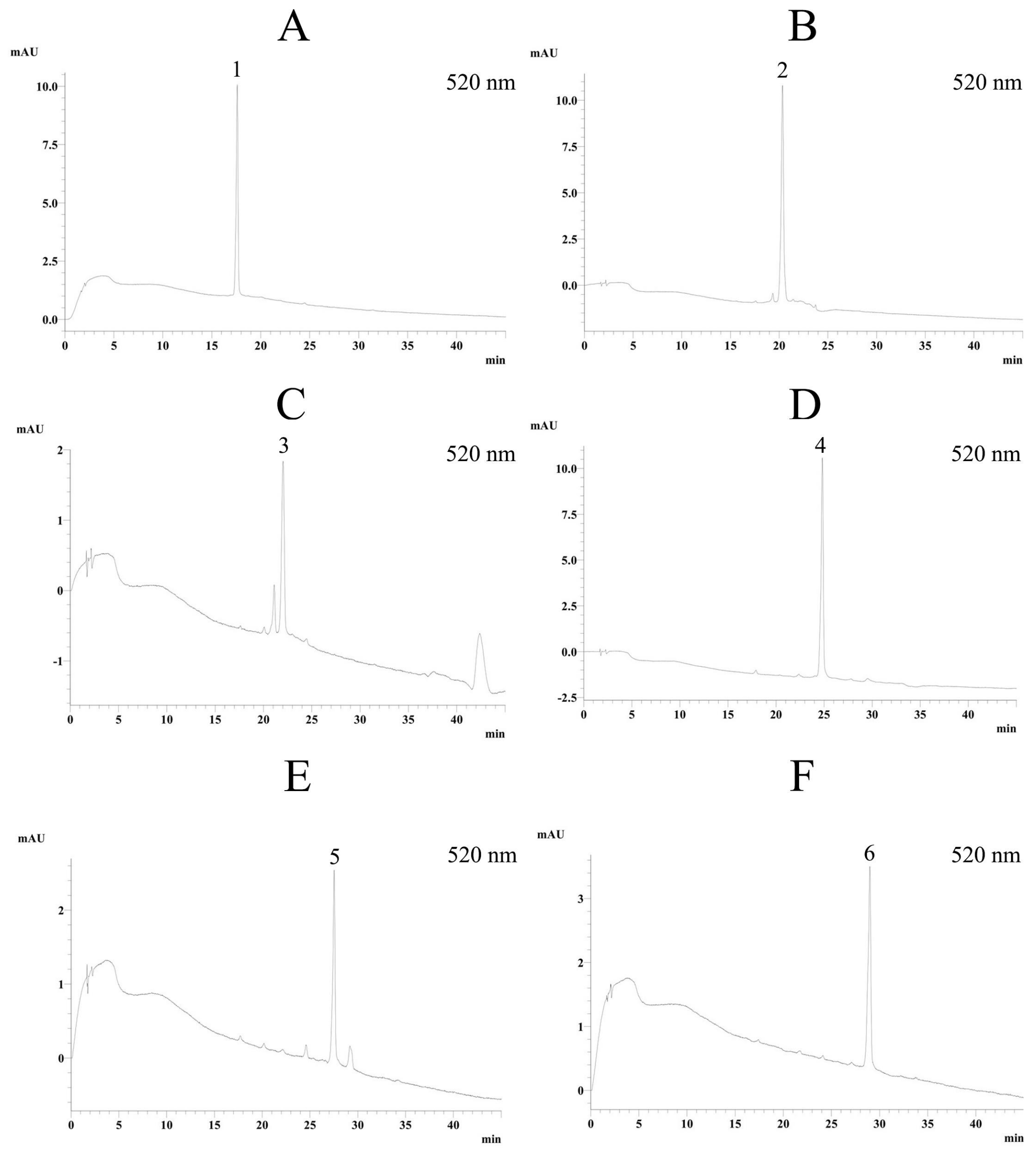

Figure S3. High-performance liquid chromatography–diode array detector (HPLC-DAD) (520 nm) chromatogram of purple corncob anthocyanin monomer, serial numbers (1~6) correspond to peak numbers in Table 1.

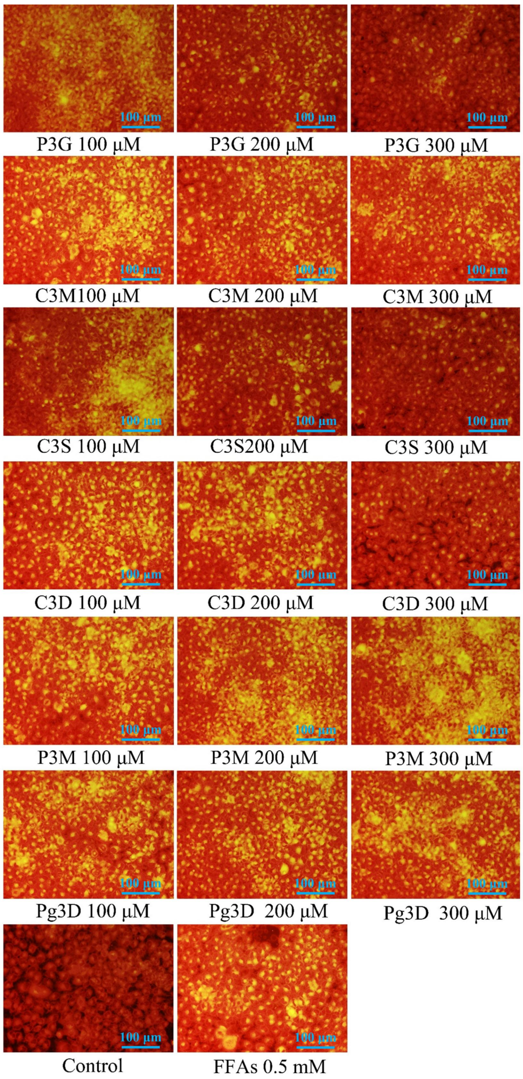

Figure S4. Effect of Purple Corncob Anthocyanidins on FFAs-induced Lipid Accumulation in L02 Cells via Nile Red Staining. P3G, peonidin-3-o-glucoside; C3M, cyanidin-3-(6'-malonylglucoside); C3S, cyanidin 3-(6"-succinyl-glucoside); C3D, cyanidin 3-o-3",6"-o-dimalonylglucoside; P3M, peonidin 3-(6"-malonyl-glucoside); Pg3D, pelargonidin 3-o-3",6"-o-dimalonylglucoside.

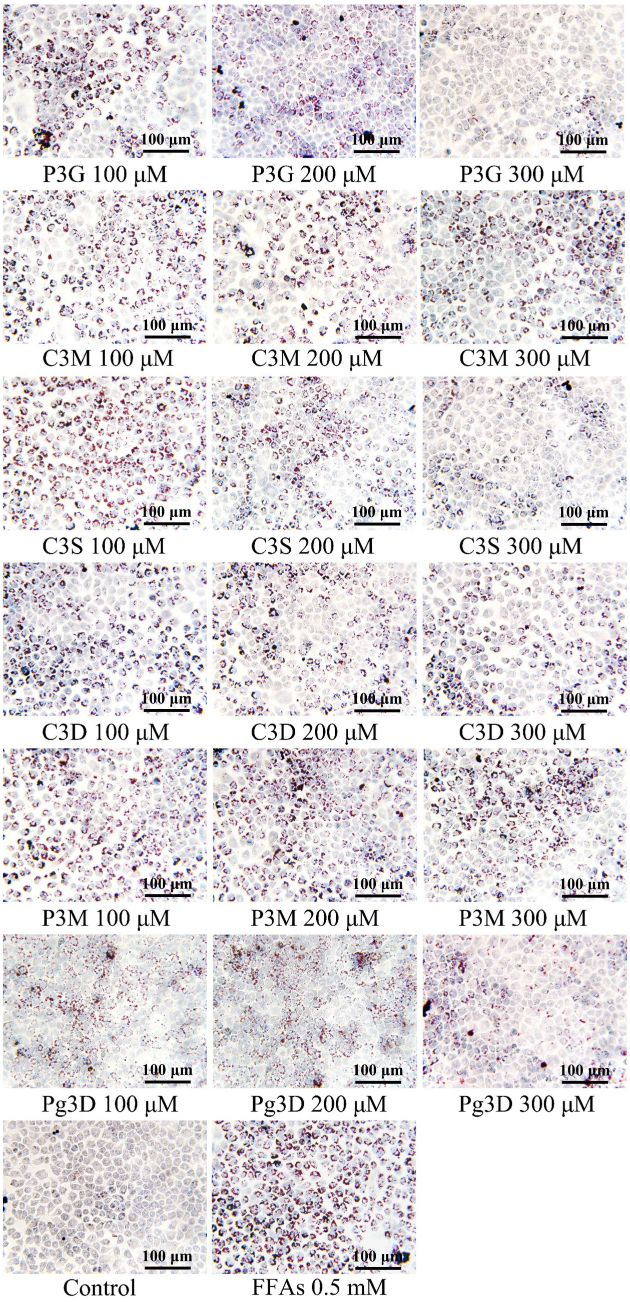

Figure S5. Effect of Purple Corncob Anthocyanidins on FFAs-induced Lipid Accumulation in L02 Cells via oil red O Staining. P3G, peonidin-3-o-glucoside; C3M, cyanidin-3-(6'-malonylglucoside); C3S, cyanidin 3-(6"-succinyl-glucoside); C3D, cyanidin 3-o-3",6"-o-dimalonylglucoside; P3M, peonidin 3-(6"-malonyl-glucoside); Pg3D, pelargonidin 3-o-3",6"-o-dimalonylglucoside.

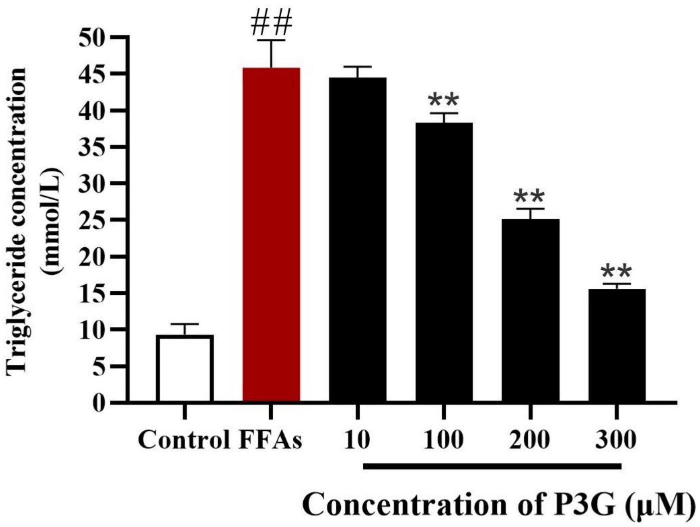

Figure S6. The intracellular Triglyceride contents in L02 hepatic cells. Following 0.5 mM FFA treatment, L02 cells were incubated with P3G at several concentrations (10, 100, 200, and 300 μM) for 24 hours. Triglyceride contents were assayed by assay kits as described in the text. Values were mean ± SD (n = 3) and expressed in mmol/L. (The control group was considered as 100%; #p < 0.05, ##p < 0.01 compared with control; *p < 0.05, **p < 0.01 vs FFA-treated group.)

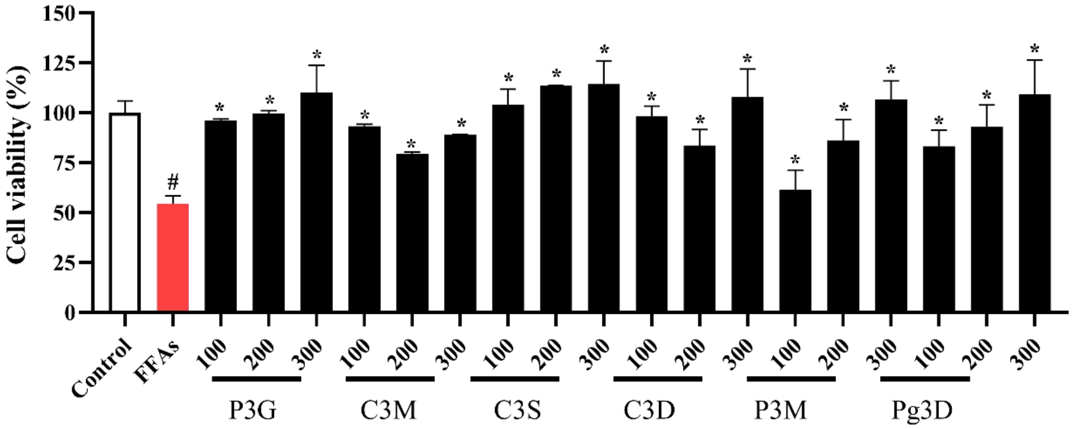

Figure S7. The effect of purple corncob anthocyanin monomers on the viability of L02 cells after treated by FFAs, control group was considered as 100%. (#p < 0.05 compared with control, *p < 0.05 vs FFAs-treated alone). P3G, peonidin-3-o-glucoside; C3M, cyanidin-3-(6'-malonylglucoside); C3S, cyanidin 3-(6"-succinyl-glucoside); C3D, cyanidin 3-o-3",6"-o-dimalonylglucoside; P3M, peonidin 3-(6"-malonyl-glucoside); Pg3D, pelargonidin 3-o-3",6"-o-dimalonylglucoside.

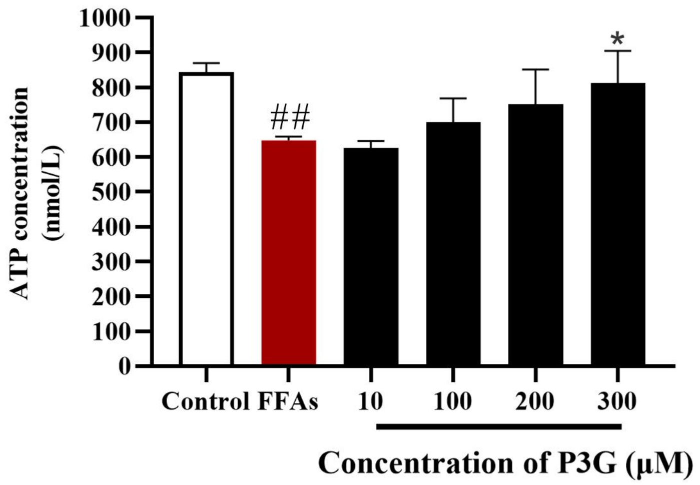

Figure S8. The ATP contents in L02 hepatic cells. Following 0.5 mM FFA treatment, L02 cells were incubated with P3G at several concentrations (10, 100, 200, and 300 μM) for 24 hours. ATP contents were assayed by assay kits as described in the text. Values were mean ± SD ($n = 3$) and expressed in nmol/L. (The control group was considered as 100%; #$p < 0.05$, ##$p < 0.01$ compared with control; *$p < 0.05$, **$p < 0.01$ vs FFA-treated group.)

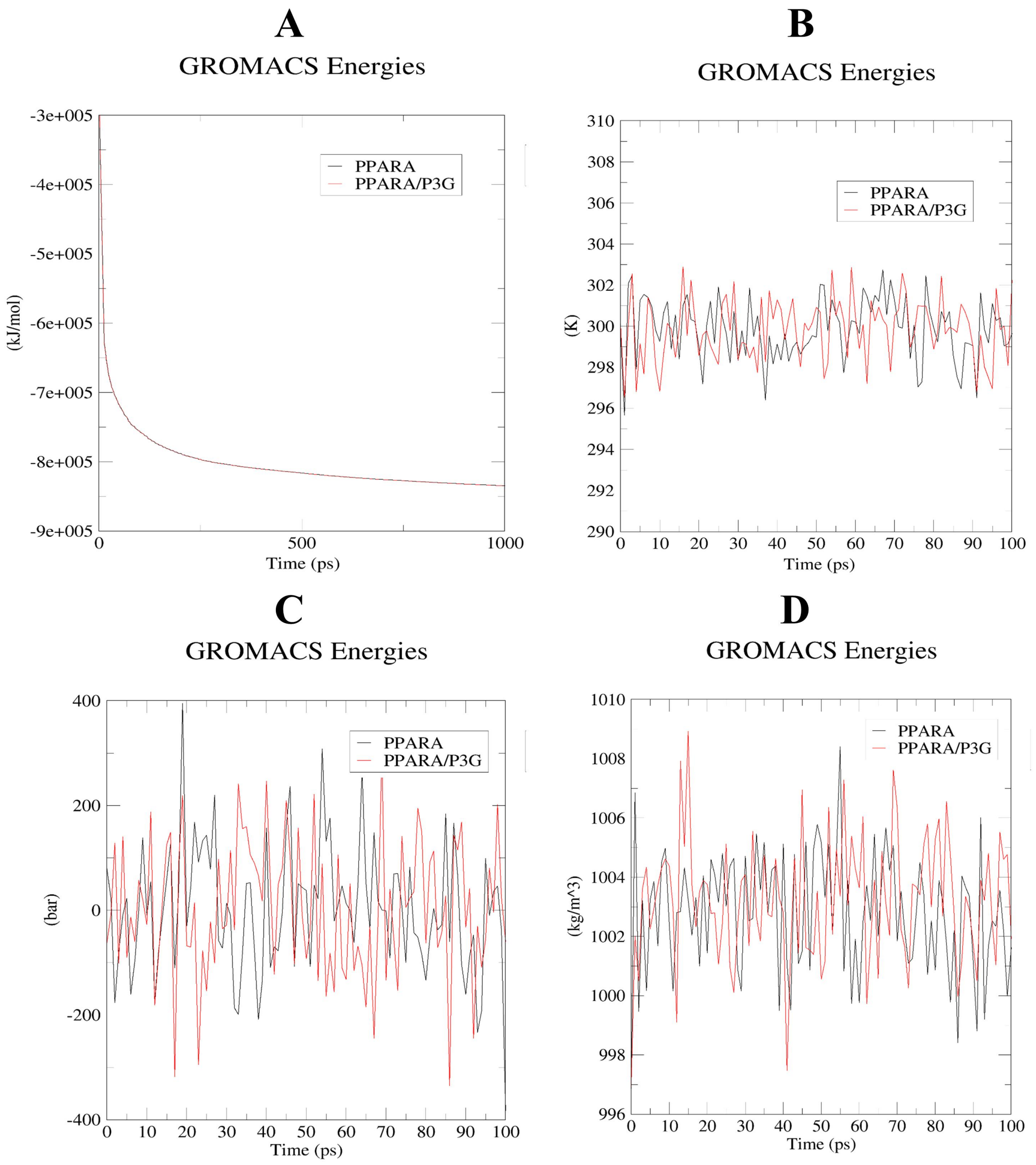

Figure S9. Evaluation of MD simulations. (A) The potential energy changes during energy minimization. (B) The temperature changes during NVT simulation. Pressure (C) and density (D) changes during NPT simulation.

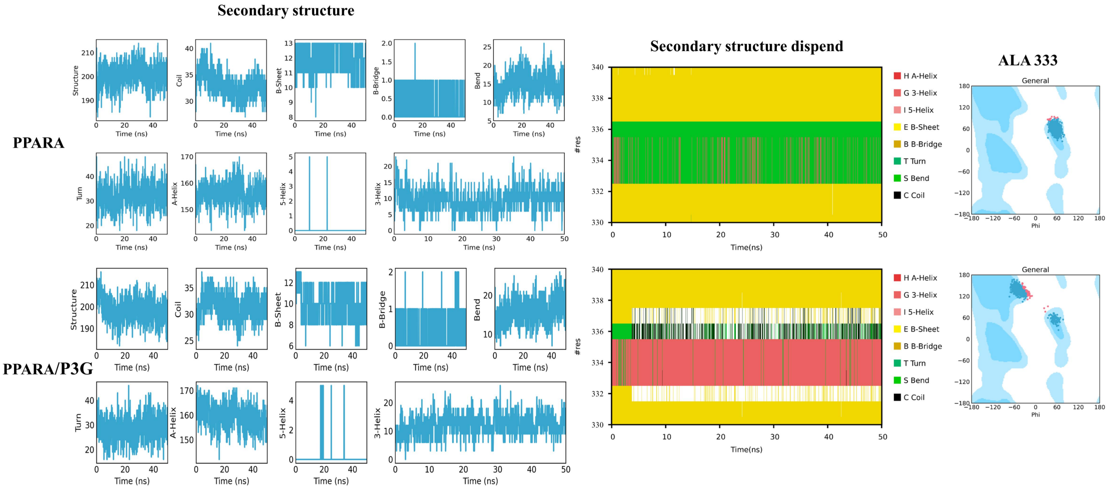

Figure S10. Ramachandran plot and secondary structure of PPARA and PPARA/P3G complex.

Raw western blot bands

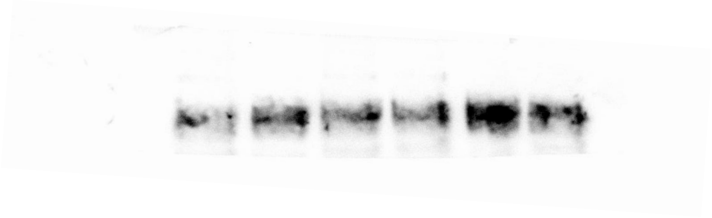

LAMP1

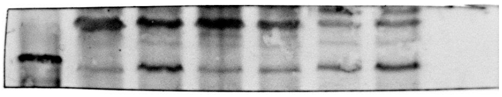

TFEB in the cytoplasm

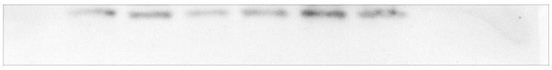

Cathepsin D

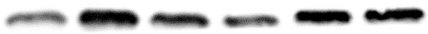

GAPDH

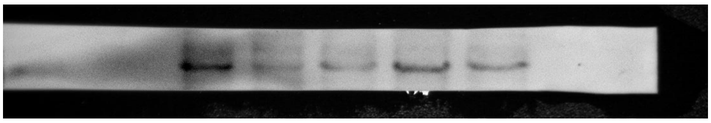

TFEB in nuclents

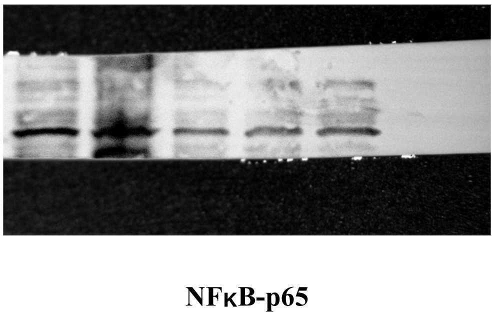

NFκB-p65

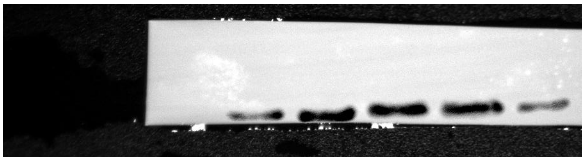

Caspase 1

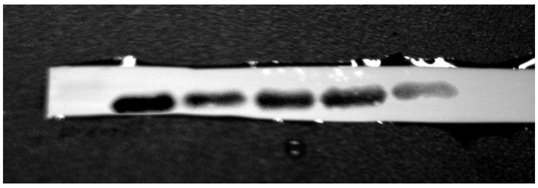

GAPDH
